# Supplementary material for: The interactome of intact mitochondria by cross-linking mass spectrometry provides evidence for coexisting respiratory supercomplexes
Source: Mol Cell Proteomics. 2017 Dec 8;17(2):216–32. doi: 10.1074/mcp.RA117.000470 (PMC5795388; doi:10.1074/mcp.RA117.000470)
Supplement: Supplemental Data [file supp_RA117.000470_133922_0_supp_23978_fzffwf.zip › spectra_annotation/mito_DR_spectra_annotation/30-1-11-1-1-1.pdf]

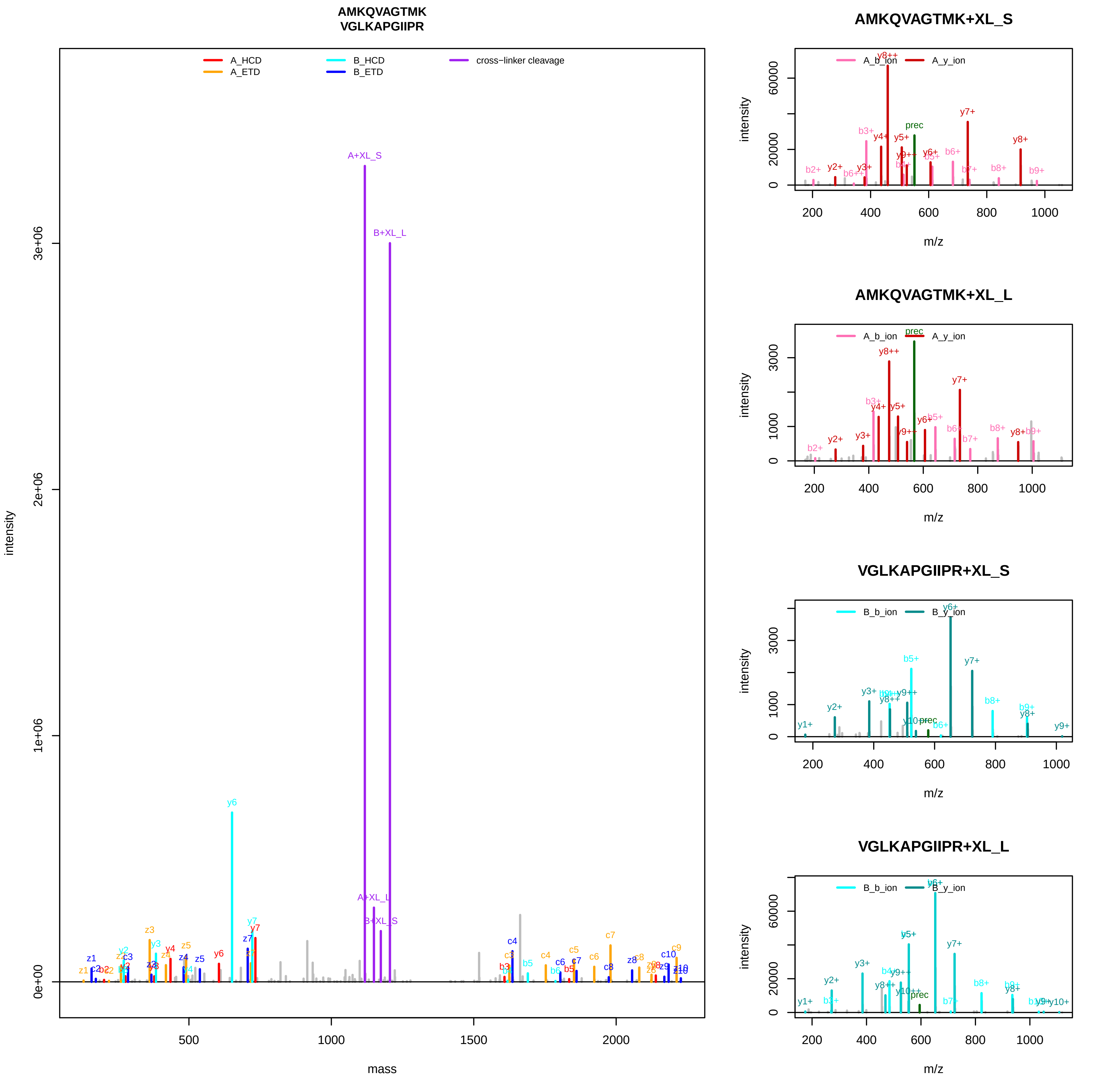
AMKQVAGTMK
VGLKAPGIIPR
A_HCD
A_ETD
B_HCD
B_ETD
cross−linker cleavage
A+XL_S
B+XL_L
A+XL_L
B+XL_S
y6
y7
z3
c7
c4
c9
intensity
mass
0e+00
1e+06
2e+06
3e+06
500
1000
1500
2000
AMKQVAGTMK+XL_S
AMKQVAGTMK+XL_L
VGLKAPGIIPR+XL_S
VGLKAPGIIPR+XL_L
A_b_ion
A_y_ion
B_b_ion
B_y_ion
prec
y8++
y7+
y8+
y6+
y5+
y4+
b3+
b5+
b8+
b9+
intensity
m/z
200
400
600
800
1000
